# Beyond geopolitics: Greenland's suicide crisis

**DOI:** 10.1016/j.lanepe.2026.101642

**Published:** 2026-03-02

**Authors:** The Lancet Regional Health – Europe

Greenland has dominated international headlines since US President Donald Trump publicly expressed interest in acquiring the territory. Yet, beyond debates over sovereignty and strategic value lies an urgent crisis. Suicide is one of Greenland's leading causes of premature death. In 2023, suicide accounted for 7.4% of all deaths in Greenland, and the territory consistently ranks among the highest for suicide rates worldwide. Despite its status as an autonomous territory of the Kingdom of Denmark, Greenland's age-standardised suicide rate of 71.3 per 100,000 people vastly exceeds that of the Nordic region, at almost 10 times the rate in Denmark. With a population of only 56,000, these statistics reflect a painful reality: most Greenlanders have lost friends or family members to suicide. But despite the increased risk of suicide in people bereaved by suicide, no national support programmes exist.

Suicide disproportionately affects young people in Greenland, with men aged 20–24 years at the highest risk, whereas suicide in Nordic countries peaks in middle-aged men. Greenland is therefore not only experiencing high rates, but also a distinct epidemiological pattern.

Around 90% of the population are Greenlandic Inuit, whose culture is rooted in connections to nature, community, interdependence, and tradition, but the territory's colonial past has contributed to drastic societal changes. Suicide rates have increased sharply since the 1970s, peaking at 120.5 per 100,000 people in the 1980s, after a period of rapid Danish led modernisation and a shift away from traditional Inuit livelihoods of hunting and fishing. This transition (including relocation from small settlements to larger towns, a move towards wage labour, and reliance on energy-dense imported foods) redefined societal roles and community structures within a generation. The effects persist in this post-colonial environment, leaving young people struggling with identity loss and intergenerational conflict. Despite this pattern, school based prevention programmes are scarce.

Socioeconomic pressures compound these structural challenges. Nearly 18.0% of the population were at risk of poverty in 2024 versus 11.6% in Denmark. Additionally, alcohol and substance misuse are common, especially in young people. The Greenland Population Health Survey reported that 35% of people aged 15–34 years engage in heavy episodic drinking. Adverse childhood experiences, including alcohol misuse, also play a role as 22% of Greenlanders with alcohol problems in their childhood home had suicidal thoughts.

Despite public funding, access to mental health services is uneven, often requiring long and impractical travel to major hospitals. Individuals who are hospitalised after a suicide attempt are offered up to ten sessions with a psychologist from the psychiatric ward in Nuuk—an infeasible treatment for many. Only a third of individuals who died by suicide in 2012–15 had contact with health-care services in the 6 months before death. There are not enough mental health professionals, although reliable data on exact numbers are scarce.

Addressing Greenland's suicide crisis requires more than clinical care; it demands a coordinated, culturally grounded effort that begins within communities and recognises the legacy of Danish led modernisation and the post-colonial period. Prevention must begin early, with investment in school based mental health literacy and resilience building and in culturally meaningful livelihoods and community programmes that strengthen connection to language, land, and traditional knowledge—protective factors that foster belonging and purpose. Postvention services for families bereaved by suicide should be implemented nationally as a priority. Mental health services and follow-up care must be expanded beyond Nuuk, with stronger data collection to guide policy and resource allocation and continued training and support for Inuit professionals to deliver culturally competent care within communities. A national alcohol strategy and action on poverty and inequality should be prioritised to tackle socioeconomic determinants of suicide. Above all, interventions must be developed in genuine partnership with Greenlandic Inuit communities, ensuring that cultural continuity and collective agency are central to public health policy.

Against this backdrop, President Trump's repeated expressions of desire to acquire Greenland are troubling. For a population with a history shaped by colonisation, suggestions of land purchase or transfer risk undermining a fundamental determinant of mental health: agency. A sense of control, belonging, and collective self-determination is central to psychological wellbeing. The notion that Greenland can be purchased or transferred ignores the foundational principle of Inuit self-determination: land is not owned but cared for. Recognising the Greenlandic Inuit as custodians of their land is essential to safeguarding cultural survival and population health.

